# Bioactive Compounds from *Kalanchoe* Genus Potentially Useful for the Development of New Drugs

**DOI:** 10.3390/life13030646

**Published:** 2023-02-26

**Authors:** Luana Beatriz dos Santos Nascimento, Livia Marques Casanova, Sônia Soares Costa

**Affiliations:** 1 SENAI CETIQT, Rua Fernando de Souza Barros, 120-Cidade Universitária, Rio de Janeiro 21941-857, RJ, Brazil; 2 Instituto de Microbiologia Paulo de Góes, Centro de Ciências da Saúde, Universidade Federal do Rio de Janeiro (UFRJ), Av. Pedro Calmon, 550-Cidade Universitária, Rio de Janeiro 21941-901, RJ, Brazil; 3 Instituto de Pesquisas de Produtos Naturais, Centro de Ciências da Saúde, Universidade Federal do Rio de Janeiro (UFRJ), Av. Pedro Calmon, 550-Cidade Universitária, Rio de Janeiro 21941-901, RJ, Brazil

**Keywords:** *Bryophyllum*, flavonoids, bufadienolides, traditional use, herbal compounds, innovation

## Abstract

The genus *Kalanchoe* Adans. (Crassulaceae) is native to Madagascar and comprises 145 species, being naturalized in the tropics and cultivated worldwide. In addition to having ornamental value, several *Kalanchoe* species are commonly used in popular medicine for the treatment of inflammatory conditions, wounds, gastric ulcers, and other diseases. The great importance of the genus is reflected on its acknowledgment by traditional and alternative health systems and organizations, as well as on the growing number of papers reporting pharmacological properties of extracts and isolated compounds from *Kalanchoe*. Among these properties, we highlight anti-inflammatory, antitumor, wound healing, antiulcer, and muscle relaxing properties. These activities are attributed mostly to flavonoids and bufadienolides, the main secondary metabolites reported in *Kalanchoe* extracts. While bufadienolides are generally related to cytotoxic and muscle relaxing activities, flavonoids are commonly reported as anti-inflammatory and wound healing agents. This review provides up to date information and perspectives on bioactive compounds from the *Kalanchoe* genus that are potentially useful for the development of new drugs. It includes not only a discussion on the advantages of the *Kalanchoe* species as source of bioactive compounds, but also the gaps, opportunities, and challenges to translate the acquired knowledge into innovation for drug development.

## 1. Introduction

The *Kalanchoe* Adans. (Syn.: *Bryophyllum* Salisb.) genus comprises succulent perennial plants intensively used for ornamental and medicinal purposes [1,2]. It belongs to Crassulaceae family and currently encompasses 145 species [3], among which some are largely known, such as *Kalanchoe pinnata* (Syn. *Bryophyllum pinnatum*), *K. daigremontiana* (*B. daigremontianum*), *K. blossfeldiana*, and *K. delagoensis* (Syn. *K. tubiflora*, *B. delagoense*, *B. tubiflorum*). The genus is native to Madagascar, although its species can be found worldwide, especially in the tropics due to easy propagation and acclimation of the plants [2,4].

Plants of the genus are widely used in traditional medicine in several countries, including India, China, South Africa and other African nations, and Brazil, as well as in alternative medicine systems. Indeed, in some of these countries, we can find reports of the use of *Kalanchoe* plants in Ayurveda, Chinese Traditional Medicine, and anthroposophic medicine [1,5,6,7]. In particular, in Brazil, *K. pinnata* is part of a list of medicinal plants to be used in the national public health system (SUS–Sistema Único de Saúde) [8]. Among the ethnomedicinal uses, plants of the genus are reputed therapeutics in the treatment of inflammatory conditions, wounds, gastric ulcers, genito-urinary disorders, and other illnesses [1,9].

**Figure 1:**
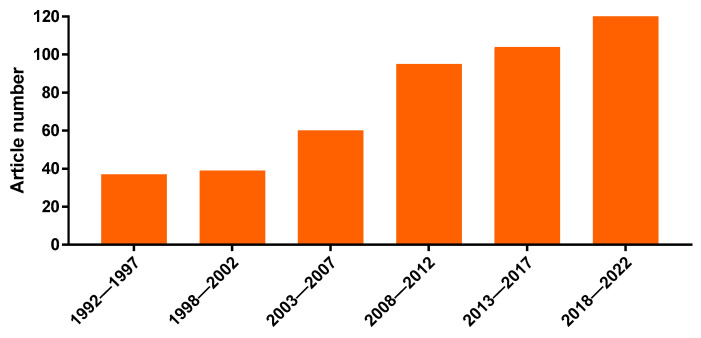
Evolution of the number of papers in indexed journals regarding medicinal uses and properties of *Kalanchoe* plants, distributed in 5-year periods (from 1992 to 2022). Source: PubMed, consulted on 15 December 2022 with the keywords “*Kalanchoe*” and “*Bryophyllum*”.

The multiple medicinal uses of *Kalanchoe* plants stimulated several pharmacological studies by research groups around the world. In fact, the importance of the genus is corroborated by the increasing number of papers regarding medicinal uses and properties of *Kalanchoe* species in the last 30 years, according to the PubMed database (Figure 1).

Several biological activities have been shown for extracts, fractions, and some isolated compounds of *Kalanchoe* plants. The most cited activities are anti-inflammatory, antimicrobial, wound-healing, muscle relaxing, and antitumor activities [1,6,9,10,11]. Bufadienolides and flavonoids are the most reported compounds in plant extracts and stand out as bioactive molecules, being potentially useful for the development of new drugs [1,6,12]. Despite this knowledge, a review focused on the importance and pharmacological properties of both bufadienolides and flavonoids is unprecedented. Therefore, this paper is a critical review of the literature (1989–2023) and provides up to date information and perspectives on these bioactive compounds, also including a discussion on the advantages of the *Kalanchoe* genus, as well as the gaps, opportunities, and challenges to translate the acquired knowledge into innovation for drug development.

Three decades of pioneering multidisciplinary studies conducted by our group and collaborators described a broad pharmacological profile of extracts and bioactive compounds, mainly flavonoids, of *Kalanchoe* species (including *K. brasiliensis*, *K. pinnata*, *K. daigremotiana*, *K. gastonis-bonnieri*, and *K. thyrsiflora*, among others) demonstrating, for example, antioxidant, anti-inflammatory, antileishmaniasis, antiviral, immunomodulatory, and anti-allergic activities [1,11,13,14,15,16,17,18]. We have also worked on biotechnological aspects of plants of this genus, demonstrating the elicitation of flavonoids production and the optimization of their extraction [19,20,21].

The papers considered in this review were obtained from PubMed, Scopus, and Google Scholar databases, resulting from the search with the following keywords combined: “*Kalanchoe*”, “*Bryophyllum*”, “flavonoids”, and “bufadienolides”. Papers reporting only the biological activity of *Kalanchoe* extracts, without identifying the bioactive compound responsible for the described activity, were not considered in detail, being only cited in some parts of the text. Additionally, some criteria were taken into account for the choice of the papers here cited, such as the quality of the information presented (based on the analytical studies, chemical characterization of the compounds, and biological assays conducted) and the indexation of the journal in which they were published.

## 2. Main Classes of Bioactive Compounds in *Kalanchoe* Species

### 2.1. Bufadienolides

Bufadienolides are C-24 steroids characterized by a six-membered lactone (α-pyrone) ring located at C-17β. They can be found in the form of glycosides and aglycones in animals (mainly toads from the genus *Bufo*) and in several plant families such as Crassulaceae, Iridaceae, and Melianthaceae. The biological activities most reported for this class of compounds are cardiotonic and antitumor activities [12,22]. Species of the genus *Kalanchoe* are known to produce bufadienolides with diverse structural features, including glycosides and orthoacetate forms. These compounds have been reported mainly in *K. daigremontiana*, *K. delagoensis* (syn.: *Kalanchoe tubiflora*), *K. pinnata*, *K. ceratophylla* (syn.: *Kalanchoe gracilis*), *K. tomentosa,* and *K. lanceolata* [12].

**Figure 2:**
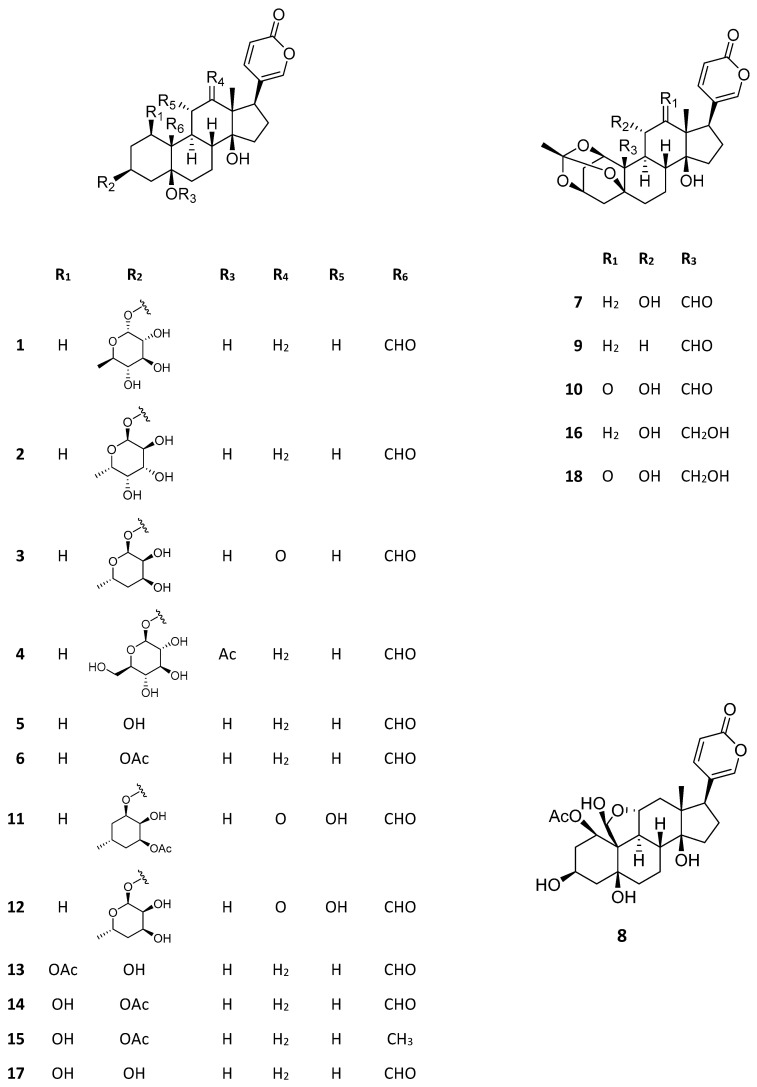
Structures of *Kalanchoe* bioactive bufadienolides. Kalanchoside A (**1**), kalanchoside B (**2**), kalanchoside C (**3**), thesiuside (**4**), hellebrigenin (**5**), hellebrigenin-3-acetate (**6**), bryophyllin A (**7**), bryophyllin B (**8**), bersaldegenin-1,3,5-orthoacetate (**9**), daigremontianin (**10**), kalantuboside A (**11**), kalantuboside B (**12**), bersaldegenin-1-acetate (**13**), bersaldegenin-3-acetate (**14**), daigredorigenin-3-acetate (**15**), bryophyllin C (**16**), bersaldegenin (**17**), bryotoxin B (**18**).

The great majority of pharmacological studies carried out with *Kalanchoe* bufadienolides are related to their antitumor properties. Bufadienolides seem to be the main substances responsible for the antitumor activity of *Kalanchoe* extracts, as evidenced by bioassay-guided studies [23,24,25,26]. Table 1 summarizes the data on antitumor properties of bufadienolides from *Kalanchoe* species in human cell lines. Their structures (**1**–**18**) are shown in Figure 2. Among these substances, bryophyllin A (7), also known as bryotoxin C, stands out. This compound, found in *K. ceratophylla*, *K. delagoensis*, and *K. pinnata*, has evidenced antitumor activity in eleven different cancer cell lines. Bersaldegenin-1,3,5-ortoacetate (**9**), also found in different *Kalanchoe* species, is also worth mentioning as well, having evidenced activity against nine distinct cell lines. The action mechanism of this substance in HeLa cells, a cervical cancer cell line, was recently evaluated in detail by Stefanowicz-Hajduk [27]. Bersaldegenin-1,3,5-ortoacetate induced DNA damages, overproduction of reactive oxygen species, and cell cycle arrest in the G2/M phase. The DNA damages were possibly associated with the overexpression of NF-κB inhibitor genes, whose pathways take part in the regulation of gene transcription [27].

Bryophyllin A, along with other two bufadienolides (kalantubosides A and B—**11** and **12**) from *K. delagoensis,* also had their activity in CL1-5 human lung cancer cells evaluated at the molecular level [28]. The substances produced no significant induction of apoptosis or cell cycle arrest and their main cell death mechanism was shown to be the activation of autophagy pathways [28].

Interestingly, in another study, kalantuboside B (**12**) was shown to induce apoptosis of A2058 melanoma cells, probably via mitochondria, death-receptor-mediated, and ER stress pathways, and the observed autophagy was probably not involved in cell death [29]. The authors also conducted further in vivo studies with A2058-xenografted mice. The in vivo experiments confirmed the previous in vitro results: kalantuboside B (0.4 mg/kg i.p. every two days for 8 weeks) was able to suppress tumor development by apoptosis promotion with no toxicity signs [29].

Other pharmacological activities are attributed to bufadienolides. A bufadienolide-enriched fraction from *K. pinnata* leaf juice revealed relaxing activity in bladder porcine muscle strips (0.1–1 mg/mL), while a flavonoid-enriched fraction from the same extract showed no significant activity [33]. In another study carried out by the same group, both bufadienolides and flavonoid-enriched fractions reduced the contractility of human myometrium [34]. Nevertheless, the former was the most active (40% decrease at 1 µg/mL), evidencing that bufadienolides are the main factor responsible for *K. pinnata* activity on muscle tissue, an important pharmacological effect in the context of the anthroposophic medicinal use of the plant for overreactive bladder and premature labor (see Section 3.1). The main bufadienolides present in the enriched fraction in both studies were bersaldegenin-1-acetate (**13**), bersaldegenin-3-acetate (**14**), bersaldegenin-1,3,5-orthoacetate (**9**), and bryophyllin A (**7**) [33,34]. A more recent study of the authors evaluated the effects of bioactive compounds from *K. pinnata* in the oxytocin signaling pathways in human myometrial cell cultures and showed that both bufadienolides and flavonoids take part in the cell events that lead to the inhibition of oxytocin signaling by *K. pinnata* leaf extracts [35]. The authors evaluated a bufadienolide-enriched fraction, a flavonoid-enriched fraction, and bersaldegenin-1,3,5-orthoacetate. All of them showed to inhibit the oxytocin-induced increase in intracellular calcium concentration, but none of them were as active as the whole leaf extract.

In addition to the studies of bufadienolides from *K. pinnata* extracts, there is also evidence for the activity of these compounds from *K. daigremontiana*. A bufadienolide-enriched fraction from a root aqueous extract of the plant showed to be active on hemostasis, being able to inhibit the thrombin activity in vitro in the concentration range of 1–50 µg/mL. A molecular docking study showed that the most probable thrombin inhibitors in the tested fraction were the bufadienolides bersaldegenin (**17**), bersaldegenin 1-acetate (**13**), bersaldegenin 1,3,5-orthoacetate (**9**), and daigredorigenin 3-acetate (**15**), as well as the lignoids hovetrichoside C and schisandriside [36]. Thrombin is a critical plasma coagulation factor and the capacity of bufadienolides to inhibit its enzymatic activity points to a possible anti-thrombotic effect [36]. Additionally, the same group evaluated the activity of a similar bufadienolide-enriched fraction from *K. daigremontiana* roots on the plasmin activity. Plasmin is part of the fibrinolytic system, which regulates the extent of formed thrombi. The type of effect exerted by the fraction on the enzymatic properties of plasmin was dependent on the concentration range: lower concentrations (0.05–2.5 µg/mL) enhanced its activity, while higher concentrations (25–50 µg/mL) resulted in inhibition. As stated by the authors, the effect observed probably resulted from an allosteric regulation of plasmin activity. According to the docking studies, the most probable bioactive compounds from the fraction were the bufadienolides bersaldegenin 1-acetate (**14**) and bryotoxin B (**18**), as well as hovitrichoside C [37].

### 2.2. Flavonoids

Flavonoids are probably the best known and most active molecules found in *Kalanchoe* species. For these phenolic substances, a broad profile of pharmacological activity in diverse targets has been reported [1]. These compounds, ubiquitously distributed in plants, are commonly found in fruits, leaves, seeds, stems, and flowers of angiosperms [38]. In *Kalanchoe*, flavonoids from leaves are the most studied [1,39]. Flavonoids comprise the largest class of polyphenols and mainly consist of two aromatic rings (A and B), united to a central heterocyclic ring (C), resulting in a basic unit C6-C3-C6. These substances are derived from mixed biosynthetic pathways: while the A-ring originates from the acetic acid pathway, with characteristic hydroxylation at 5′ and 3′ positions, the B-ring originates from the shikimic acid pathway [38]. Flavonoids comprise different structural subclasses such as flavonols, flavones, flavanonols, flavanones, flavanols, and anthocyanidins, differentiated mainly by the degree of oxidation and substituents of the C ring [40]. These polyphenolic compounds have well known biological activities, several of them related to their ability to scavenge free radicals, resulting in antioxidant activity. These include the prevention of cardiovascular and neurodegenerative diseases, cancer, inflammatory processes, atherosclerosis, and in their action in diabetes and autoimmune diseases, as well as antimicrobial and antiviral properties [41]. These molecules are also known for their immunomodulatory activity [42].

Flavonoids have been extensively reported in different *Kalanchoe* species, including *K. brasiliensis*, *K. ceratophyla* (syn.: *K. gracilis*), *K. spathulata*, *K. pinnata*, *K. daigremontiana*, *K. blossfeldiana*, *K. gastonis-bonnieri,* and others [1,17,39]. These compounds were firstly identified in the genus in the 1970s with the studies of Gain and Gupta [43], who showed the presence of quercetin and kaempferol glycosides in *K. pinnata* leaf extracts. Indeed, studies developed in the last 50 years have shown that flavonoids in this genus are mainly represented by flavonol and flavone glycosides [1,43,44]. Despite the growing number of studies describing pharmacological actions of *Kalanchoe* extracts, and the knowledge about flavonoids as important chemical constituents in this genus, most of the studies have just reported the presence of these molecules, without properly investigating their bioactive behavior. Indeed, several of these studies only suggest the potential role of flavonoids in a given activity [45,46,47,48,49,50,51,52], without assessing the isolated compound to confirm its bioactivity. The characterization of bioactive compounds is important not only to define the molecules responsible for a given biological property, but also for the development of phytomedicines. The use of such substances as biomarkers (active marker) for standardization and quality control guarantees the safety and reproducibility of the pharmacological effects of these formulations, being preferable to the use of analytical markers, unrelated to the therapeutic activity [53].

Due to the importance of identifying the bioactive compound, the number of papers tentatively attributing the biological activity of *Kalanchoe* extracts to flavonoids or to flavonoid-enriched fractions is increasing nowadays. In a study on the activity of *K. pinnata* in a mice model of lupus arthritis, the authors observed that the ethyl acetate fraction (rich in quercetin glycosylated flavonoids), obtained from a leaf aqueous extract, inhibited the progression of the disease [54]. This fraction also produced positive effects on anti-Smith antibody and T reg in lupus mice [55]. Still regarding *K. pinnata*, ethyl acetate fractions enriched in quercetin, luteolin, isorhamnetin, and luteolin-7-glucoside ameliorated oxidative imbalance in neurotoxicity caused by aluminum chloride in rats, showing a neuroprotective effect [56]. Antioxidant properties and anticholinesterase activity have also been described for this fraction [57]. A chloroform fraction of stem methanolic extracts from *K. ceratophyla* (syn. *K. gracilis*) presenting a high content of flavonoids showed antioxidant, anti-inflammatory, and antiproliferative activities [58]. In some of these studies, the effect of the whole extract is higher than that of the fractions [35,58], indicating a synergistic effect of these compounds.

**Figure 3:**
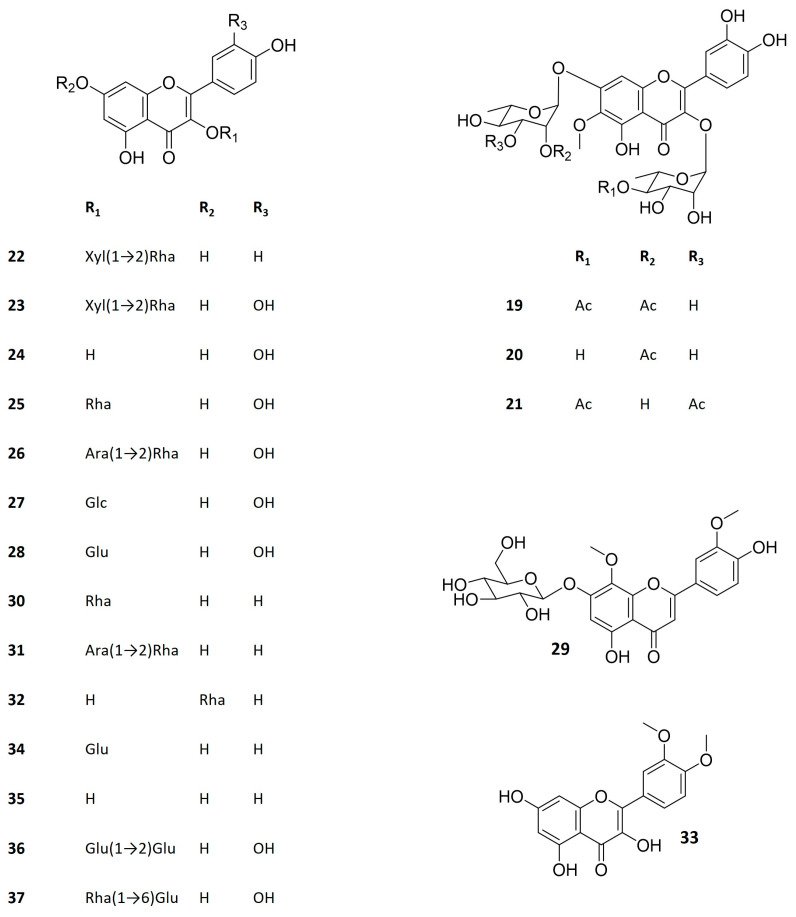
Structure of *Kalanchoe* bioactive flavonoids. Kalambroside A (**19**), kalambroside B (**20**), patuletin 3-*O*-(4″-*O*-acetyl-α-L-rhamnopyranosyl)-7-*O*-(3‴-*O*-acetyl-α-L-rhamnopyranoside) (**21**), kaempferol 3-*O*-β-D-xylopyranosyl (1→2) α-L-rhamnopyranoside (**22**), quercetin 3-*O*-β-D-xylopyranosyl (1→2) α-L-rhamnopyranoside (**23**), quercetin (**24**), quercitrin (**25**), quercetin 3-*O*-α-L-arabinopyranosyl (1→2) α-L-rhamnopyranoside (**26**), miquelianin (**27**), isoquercitrin (**28**), 4′,5-dihydroxy-3′,8-dimethoxyflavone-7-*O*-D-glucopyranioside (**29**), afzelin (**30**), kapinnatoside (**31**), α-rhamnoisorobin (**32**), 3′,4′-dimethoxy quercetin (**33**), astragalin (**34**), kaempferol (**35**), quercetin-3-*O*-sophoroside (**36**), rutin (**37**). Ac–acetate, Ara–arabinose, Glc–glucuronic acid, Glu–glucose, Rha–rhamnose, Xyl–xylose.

Several examples of bioactivity for flavonoids isolated from extracts of *Kalanchoe* plants are presented in Table 2. Their chemical structures can be seen in Figure 3. In particular, previous studies developed by our group demonstrated several activities of flavonoids isolated from *Kalanchoe* species, such as leishmanicidal, anti-allergic, antinociceptive, antiedematogenic, anti-inflammatory, antiviral, antitumor, immunosuppressive, and wound healing activities [1,11,15,16,59,60,61,62].

Among the flavonoids described in *Kalanchoe* genus, the glycosyl derivatives of quercetin, kaempferol, eupafolin, and patuletin are the most common [1,39]. Considering the bioactive flavonoids in the genus, quercetin glycosides are the most cited (Table 2). Indeed, several *Kalanchoe* species have quercetin derivatives as the compounds responsible for antimicrobial (including antiprotozoal), antiviral, anti-inflammatory, antioxidant, gastroprotective, immunomodulatory, and wound healing activities (Table 2). Quercetin (**24**), quercetin 3-*O*-ramnopyranonoside (hereafter called quercitrin, **25**), and quercetin 3-*O*-α-L-arabinopyranosyl (1→2) α-L-rhamnopyranoside (hereafter referred as QAR, **26**) occurring in *K. pinnata*, the most studied species of the genus, are intensively described as bioactive compounds for several biological effects, some of them proven by in vivo assays (Table 2).

In fact, *K. pinnata* is the species for which we have the largest number of in vivo studies.

Quercetin (**24**) is a flavonol widely present in plants. Several quercetin-derived glycosides can be found in nature, varying from very common compounds, such as rutin and quercitrin, to unusual ones. The glycosylation usually occurs in the -OH at the C-3 position, but it can occur on 4′-OH and 7-OH as well [74,75]. Studies with *K. pinnata* reported that quercetin has shown antiviral, antileishmanial, immunomodulatory, and immunossupressive activities [16,60,61,63]. In addition to these effects, quercetin and other flavonoids obtained from *K. prolifera* showed antitumor activity against murine leukemic cells [71]. Studies have already revealed the wide range of quercetin activity in inhibiting the proliferation of cancer cells through different mechanisms of action [76]. Regarding human leukemia, quercetin has shown activity using a xenograft model of HL60 human leukemic cells. There is evidence that quercetin induces signaling at the levels of apoptosis, cell cycle, and autophagy culminating in the inhibition of HL60 tumor growth [77]. A pilot clinical study carried out in patients with chronic lymphocytic leukemia/small lymphocytic lymphoma to evaluate the antitumor effect of quercetin (500 mg/twice daily; 3 months) showed promising results despite the small sample size [78].

There is a huge number of studies supporting the antiviral properties of quercetin. In particular, this flavonoid showed inhibition against adenovirus, herpes simplex virus, Japanese encephalitis virus, and respiratory syncytial virus [74,79]. The mechanisms of action have been also described for several viruses. For instance, quercetin interacts with the NS3 helicase, NS5B polymerase, and p7 proteins of hepatitis C virus (HCV), suggesting that the anti-HCV activity of this flavonoid occurs through inhibition of NS3 helicase and heat-shock proteins [74,80,81]. Quercetin may inhibit the viral infection of the influenza virus in different ways: (i) blocking the endocytosis of the virus by the host cell, (ii) hampering the replication and translation of the virus, and (iii) increasing the viral clearance. It is worth mentioning that, recently, quercetin has been also investigated for its effect on coronavirus (Coronaviridae) [74].

Quercetin, along with some of its glycosides, also plays a significant role in the immunomodulatory activity of *K. pinnata*. This flavonoid, compared with its glycosides quercitrin, QAR, miquelianin, and isoquercitrin, exhibited the highest in vitro inhibitory activity (IC_50_ = 2.5 μg/mL) of lymphocyte proliferation in mice, suggesting that the increased glycosylation reduces the inhibitory activity under in vitro conditions [16]. In addition, another study with quercetin obtained from *K. pinnata* showed that this flavonoid inhibited degranulation and cytokine production of bone marrow-derived mast cells in in vitro assays [61]. Quercetin also exhibited a decrease in the development of airway hyperresponsiveness, airway inflammation, goblet cell metaplasia, and production of the cytokines IL-5, IL-13, and TNF in an in vivo model [61].

Quercetin has also been shown to be a molecule with antileishmanial properties. Oral treatment with this flavonoid suppressed the burden of *Leishmania amazonensis* by 76% compared with untreated animals, a result superior to that of the positive control [60]. The effect of quercetin on cutaneous leishmaniasis was compatible with its effectiveness in the visceral form of this disease [82]. Previous studies showed that quercetin has in vitro leishmanicidal activity against promastigote forms of *L. amazonensis* as well as amastigote forms of *L. amazonensis* and *L. donovani* [83,84]. Recently, it was reported that quercetin is also active against the proliferation of *L. braziliensis* promastigotes [83]. The mode of action of this flavonoid against leishmaniasis and other protozoal parasites is the matter of several studies. It is believed that quercetin acts in different ways: disrupting the mitochondria function; inhibiting heat shock proteins, DNA topoisomerases, and kinases; increasing ROS and RNS production; causing DNA degradation; or by stimulating nitric oxide and cytokines production [85].

The in vitro antileishmanial activity observed for *K. pinnata* extracts was not only attributed to quercetin. Other leishmanicidal flavonoids were isolated from the aqueous leaf extract of this *Kalanchoe* species and tested both in vitro [59,66] and in vivo [60]. These flavonoids include quercitrin (**25**), QAR (**26**), kapinnatoside (**31**), afzelin (**30**), and 4′,5-dihydroxy-3′,8-dimethoxyflavone-7-*O*-D-glycopyranoside (**29**). Kapinnatoside and afzelin, both kaempferol derivates, showed to be less active than quercetin glycosides in this study, suggesting the importance of the B ring hydroxylation feature for antileishmanial activity [59].

Despite the importance of the various biological activities demonstrated for these flavonoids obtained from *K. pinnata*, maybe the most prominent bioactive flavonoid is QAR (**26**). It is the major component of the aqueous leaf extracts of *K. pinnata*, equivalent to about 65% of the total area of chromatographic signals corresponding to phenolics detected by high performance liquid chromatography (HPLC) [19,21,67,69]. Although this compound occurs in such high content in *K. pinnata* leaf extracts, it is not a common molecule in nature (see Section 3.2), and for this reason it can be proposed as a chemical marker of this *Kalanchoe* species [21]. It is also reported in extracts of flowers of *K. pinnata* and *K. blossfeldiana* [16,86], but in lesser amounts. Considering the several biological activities of *K. pinnata* extracts attributed to this flavonoid in the last 15 years (Table 2), this compound might be proposed as bioactive marker of the species, in addition to the already attributed role of a chemical marker.

In addition to the antileishmanial activity [59,60], QAR has demonstrated several other biological activities both in vitro and in vivo [15,16,19,67,68,69]. It has exhibited a potent antioxidant activity and the inhibition of T-cell proliferation [16,19]. Furthermore, this flavonoid has shown strong anti-inflammatory (carrageenan-induced leukocyte migration test, ID_50_ 2.0 mg/kg), antiedematogenic (in croton oil-induced ear edema, ID_50_ 0.76 mg/kg), and antinociceptive (in the acetic acid-induced writhing model, ID_50_ 9.4 mg/kg) activities in mice [15]. The evaluation of the mechanism of action involved in these effects revealed that QAR inhibits the activity of both COX-1 and COX-2, with preferential inhibition to COX-1, besides reducing the TNF-α concentration in pleural exudates. These results indicate that the anti-inflammatory effects of QAR involve COX-1/COX-2 and TNF-*α* synthesis/release inhibition [15].

Also regarding the anti-inflammatory activity, QAR has recently shown in vitro inhibition and high selectivity of the PDE4B enzyme (phosphodiesterase 4B), being proposed as an interesting candidate for the design of other PDE4B inhibitors [67]. In fact, PDE4B has lately been identified as a major cyclic AMP (cAMP)-metabolizing enzyme in inflammatory cells. Therefore, the inhibition of PDE4B decreases the inflammatory responses in multiple cell types [87]. QAR is also referred to as a modulator of Organic Anion Transporting Polypeptides (OATPs) [88,89], sodium-dependent membrane carriers present in several human organs responsible for mediating the uptake of various anticancer drugs, including those used in the treatment of liver cancer [89].

Studies on the activity of *K. pinnata* in an animal model of gastric ulcer revealed the beneficial effect of ethyl acetate fractions of leaf hydroethanolic extracts. Gastroprotective activity was attributed to QAR and quercitrin [49,52]. Recently, De Araújo et al. [69] demonstrated the gastroprotective property of QAR against gastric lesions induced by ethanol and indomethacin in mice. The authors observed that the oral pretreatment with QAR (5 mg/kg) reduced the gastric lesions of the animals. However, it was supposed that this compound acts synergistically with other compounds increasing the gastroprotection [69]. It is also important to highlight that QAR and quercitrin have shown inhibitory activity in α-glucosidases, being proposed as candidates for the treatment of type II diabetes [65]. Indeed, inhibitors of α-glucosidase stand out as a noninvasive treatment for this metabolic disorder, and quercetin and its glucosides are being studied as potential drugs [90,91].

In a recent study developed by our group, a cream formulated with QAR showed a wound healing effect in a rat model of an excisional wound [68]. Creams obtained with *K. pinnata* leaf extracts and the isolated flavonoid (QAR) exhibited 95.3 ± 1.2% and 97.5 ± 0.8% of healing on the 12th day of experiment, respectively, both showing to be as effective as the positive control (a commercial cream containing neomycin and bacitracin, 96.7 ± 0.8%). Based on these results, QAR can be considered as the bioactive substance for the wound healing effect, since it plays a fundamental role in this activity [68]. Although the mode of action of the cream containing QAR has not been described, several previous works supported the wound healing effect of *K. pinnata* extracts rich in this flavonoid [92,93]. The already proven anti-inflammatory activity of QAR, in addition to the antioxidant and antimicrobial activities, can also favorably contribute to the healing stages [68]. In addition, a gel formulated with leaf extracts of *K. pinnata* and having QAR as the main compound also stimulated skin wound healing in rats in a very recent study [94]. The authors evaluated the stability of the gel by checking the content of QAR for 30 days, using this flavonoid as a chemical and quality control marker [94]. All these findings show the importance of QAR as a bioactive molecule in the wound healing effect. Quercetin and its glycosides are intensively studied as wound healing compounds and the ability of this flavonoid to improve the healing process seems to be associated with its well-established anti-inflammatory and antioxidant activities. Some other examples of quercetin derivatives with wound healing effects include quercetin-3-*O*-glucoside (isoquercitrin), quercetin-3-*O*-rhamnosyl-(1→6)-glucoside, quercetin-3-*O*-galactoside, and quercetin-3-*O*-rhamnoside (quercitrin), some of them also described in extracts of *K. pinnata* [95,96,97].

Compared to quercetin derivatives, kaempferol glycosides are less described as bioactive constituents of *K. pinnata* extracts, despite being also reported in this species and others belonging to the same genus. For instance, afzelin (**30**), a kaempferol rhamnoside found in many *Kalanchoe* species, has presented antitumor and antileishmanial activities. In the study of Aisyah et al. [71], the cytotoxic activity of afzelin was comparable with that of other kaempferol and quercetin monoglycosides, as depicted at Table 2. The in vitro effect of the flavonoid on *Leishmania amazonensis* amastigotes, on the other hand, was weaker than that presented by quercetin glycosides (QAR and quercitrin) [59]. Afzelin is a rather common flavonoid, which has been reported in a variety of species such as *Sedum dendroideum* (Crassulaceae) [98], *Bauhinia forficata* (Fabaceae) [99], *Ficus palmata* (Moraceae), and *Nymphaea odorata* (Nymphaeaceae) [100], among others. Possibly for that reason, the pharmacological properties of the flavonoid have been extensively studied. Besides the above-mentioned activities, other examples include in vivo anti-asthmatic [100] and diuretic effects [99]. The antitumor activity of the flavonoid was also demonstrated in vivo in Ehrlich’s ascites carcinoma [101] and in vitro in AGS gastric cancer cells [102], as well as in androgen-independent PC-3 and androgen-sensitive LNCaP prostate cancer cells [103]. Contrarily to afzelin, kapinnatoside (**31**) is an uncommon kaempferol derivative, which bears the same glycosylation pattern of QAR. It has also shown antileishmanial activity, but here again, the effect was weaker than that of its quercetin counterpart (QAR) [59].

## 3. Why Choose *Kalanchoe*? Singular Aspects of the Genus as a Source of Bioactive Compounds

After presenting the detailed aspects of the main bioactive compounds detected in *Kalanchoe* species, in this section, we will present the main reasons why plants of this genus can be considered a prominent source of bioactive molecules for drug development.

### 3.1. Established Uses and Traditional Knowledge: Kalanchoe Species Are Widely Used around the World

It is a fact that *Kalanchoe* plants have several medicinal uses around the world. Species of this genus are applied to treat a wide range of diseases and heath conditions. The uses of *Kalanchoe* plants include mostly infusions, cataplasms, juices, or even compresses from crushed leaves. In addition, some *Kalanchoe* species are also consumed as salads [1,39,104]. *Kalanchoe* species are intensively cultivated in home gardens, and are also found in medicinal herb markets, which can also explain their large use [1].

Among the several medicinal *Kalanchoe* species, the most used in traditional medicine is *K. pinnata*. Undoubtedly, this fact justifies the greater interest of researchers and, consequently, the greatest number of studies of this medicinal species. For the treatment of wounds, people use local leaf compresses, while oral administration is used for treating other health problems. Some *Kalanchoe* species have had their pharmacological properties evidenced in in vitro studies and even in animal models, reinforcing the traditional knowledge.

While *Kalanchoe* plants have been widely used in traditional medicine in India, Sri Lanka, Pakistan, China, African countries, and Central and South America for a long time [48,105,106,107,108,109], their use in Europe is more restricted to anthroposophic medicine. *K. pinnata* and *K. daigremontiana* (*B. pinnatum* and *B. daigremontianum*) are used in this alternative medicinal system, and both species are listed in the German Homeopathic Pharmacopoeia (HAB) 2014 [6]. In particular, *K. pinnata* has been prescribed by practitioners of anthroposophic medicine for a wide range of health problems such as insomnia and emotional disturbances, and to prevent premature labor since 1970 [6,110]. Recently, this practice has also been adopted in perinatal centers in Switzerland [6,111,112,113], which is important, because new tocolytic agents without side effects are strongly needed. For this reason, we can observe significant advances in preclinical and clinical studies of *K. pinnata* extract regarding its tocolytic effect [35,114,115,116]. In vitro studies have shown that flavonoids and especially bufadienolides are implicated in the tocolytic effect of *K. pinnata*, as mentioned in Section 2.1 [34,35].

*K. pinnata* also plays a prominent role as a medicinal plant in Brazil due to its range of therapeutic properties. The species was included in a list of 71 medicinal plants elaborated by the Brazilian Ministry of Health in 2009: the National List of Plants of Interest to the Unified Health System (RENISUS) [8]. This list aims to guide studies and research on medicinal plants already used in the Brazilian primary care of the Unified Health System (Sistema Único de Saúde–SUS). This Brazilian Health policy intends to provide advances in knowledge of their safety and efficacy, as well as to promote the development of herbal medicines by the pharmaceutical sector. In fact, besides the intensive popular use, there is already a great amount of knowledge about the therapeutic properties of *K. pinnata*, as well as about the mechanisms of pharmacological action in several targets, and the safety profile of extracts and formulations [15,61,62,68,117,118,119], which can, at least partially, explain the interest in this species by the Brazilian government. The set of pharmacological data about *K. pinnata* shows the potential of this medicinal plant for the development of phytomedicines as a strategic source of new bioactive substances.

### 3.2. Presence of Unusual Compounds: Kalanchoe Is a Source of Rare Compounds in Nature

As discussed throughout this review, the pharmacological activities of *Kalanchoe* species are mainly attributed to flavonoids and bufadienolides [1,6,12] (Table 1 and Table 2). Bufadienolides are compounds with limited distribution among plants [12,120]. Thus, not surprisingly, many of the bioactive bufadienolides reported in *Kalanchoe* were, up to now, described exclusively in species belonging to the genus, according to Scifinder and PubChem databases. This is the case of kalanchosides A-C (**1**–**3**), bryophyllins A and B (**7** and **8**), daigremontianin (**10**), kalantubosides A and B (**11** and **12**), daigredorigenin-3-acetate (**15**), bryophyllin C (**16**), and bryotoxin B (**18**).

On the other hand, flavonoids are secondary metabolites with a wide occurrence in plant kingdom, as previously mentioned. As discussed here, several studies on different *Kalanchoe* species showed that flavonoids (mainly quercetin skeleton), usually glycosylated at positions 3 and/or 7 with glucose and rhamnose residues, play an important role on several therapeutic targets (Table 2, Section 2.2). Among the bioactive flavonoids of *Kalanchoe* species (Table 2), most of them occur in other botanical genera, in greater or lesser amounts (e.g., quercetin, rutin, quercitrin, isoquercitrin, miquelianin, quercetin-3-*O*-sophoroside, 3′,4′-dimethoxy quercetin, kaempferol, afzelin, astragalin, and α-rhamnoisorobin). However, some other flavonoids seem to be restricted to this genus or occur in a greater amount in *Kalanchoe* plants when compared with other species, at least according to the information found in the literature so far.

Glycosylation of common aglycones with diverse sugar moieties through different inter-glycosidic linkages increases the variety and complexity of glycosylated flavonoid structures. Therefore, from a same aglycone, a less common flavonoid can be obtained depending on the uniqueness of the sugar in the glycosylation and, if there is a second or a third sugar in the structure, on the type of inter-glycosidic bond between these units. For this reason, several bioactive flavonoids found in *Kalanchoe* species have a more restricted occurrence, and some of them can be considered rare, especially when pentose units (xylose and arabinose) are the sugars linked to the aglycone. This is the case of QAR (quercetin 3-*O*-α-L-arabinopyranoside-(1→2)α-L-rhamnopyranoside—**26**), the most abundant flavonoid in aqueous extracts from *K. pinnata* with several in vitro and in vivo biological activities [15,60,68,69] (see Section 2.2).

Although QAR has a quercetin skeleton, which is quite common, the glycosylation pattern with an arabinosyl residue at position 3 of this flavonoid could explain its sparse frequency in nature. This glycosylation motif (3-*O*-arabinosyl) in a flavonol is less frequent than the glycosylation with glucosyl or rhamnosyl moieties. In fact, the enzyme flavonol-3-*O*-arabinosyltransferase contributes less to the glycosylation of flavonols than 3-*O*-glucosyl and 3-*O*-rhamnosyltransferases, as reported in *Arabidopsis thaliana* leaves [121]. This rare flavonoid has been reported in a few other plant species such as *Alphitonia philippinensis* (Rhamnaceae), *Zizyphus jujuba* (Rhamnaceae), and *Rollinia emarginata* (Annonaceae) [88,89,122]. As discussed previously here, the restricted occurrence in the plant kingdom and its relative abundance in *K. pinnata* make this flavonoid a good chemical marker for this plant species with high therapeutic potential [66].

A new and minoritarian kaempferol diglycoside–kapinnatoside (**31**), bearing the same carbohydrate moieties at the 3 position as in QAR, was also isolated from *K. pinnata*, along with another unusual flavone glycoside, 4′,5-dihydroxy-3′,8-dimethoxyflavone 7-*O*-beta-D-glucopyranoside [66]. Both compounds are also not commonly found in other plant species.

Besides flavonoids and bufadienolides, there are other molecules that can be cited as unusual in *Kalanchoe*. For instance, a glycosylated aryltetralin lignan presenting an unprecedented structure and exhibiting inhibitory activity against HSV-1 and vaccinia viruses was reported in *K. pinnata* [123,124]. The isolation of this polyphenol in this species seems to be the only report in the genus, although lignans have been detected in the aqueous extract of *K. gastonis-bonnieri*, potentially useful to treat primary benign prostatic hyperplasia [125]. Lignans are more common in *Rhodiola* species, another medicinal genus of Crassulaceae [126].

### 3.3. Kalanchoe Plants: The “Mother of Thousands” Genus Is Becoming Biotechnological

About 25% of the annual global market of medicines is based on plant molecules [127], which stimulates scientists to research and describe new bioactive ingredients obtained from this natural source [128,129]. To successfully develop phytomedicines, it is necessary not only to choose the plant source based on its activity (supported by the traditional use and scientific research) but also based on the easiness of access to the plant material. In fact, accessing sufficient material to isolate, characterize, and apply the bioactive compounds is challenging [130]. Nowadays, diverse biotechnological and cultivation techniques are being developed and applied to ensure the access to the raw material, as well as the best ways to produce, extract, and isolate their bioactive ingredients [130]. Regarding the cultivation and biotechnological knowledge for triggering the production of bioactive compounds, *Kalanchoe* plants are moving to promising places.

Apart from the sexual reproduction commonly found in superior plants (by means of flower production, cross-pollination, and seed dispersal), *Kalanchoe* has evolved a wide range of asexual reproductive strategies, mainly based on both organogenesis and embryogenesis processes localized on the leaf margin [131]. These leaf areas commonly have buds, which contribute to the easiness and wide spreading of *Kalanchoe* species in several regions, mainly in mild-climate parts of the world. Even the name of the genus refers to its facility of propagation, representing the phonetic transcription of the term in Chinese “Kalan Chauhuy” that means “which falls and grows” [132]. Indeed, *Kalanchoe* plants are often propagated from seed, leaf-cuttings, buds, and even from detached/injured leaves [132]. Plants of these genus also require low agricultural input for their cultivation, needing neither a specific type of soil nor high water availability [132]. In addition, as succulents, *Kalanchoe* plants are able to survive for long periods under environmental stresses, by accumulating water in their leaves and by producing secondary metabolites that contribute as antioxidants, radiation-protectors, and/or light-catchers [20,132,133,134,135].

Some genetic studies and breeding programs have been developed with *Kalanchoe* plants in the recent years [132,136]. It can be attributed to the horticultural importance of some species of the genus (e.g., *K. blossfeldiana*) and to the interest of plant physiologists in understanding and manipulating the Crassulacean Acid Metabolism (CAM) as well as its asexual reproduction [137,138]. The scientists are also excited to understand the evolutionary relationship between the different species of the genus. As a matter of fact, DNA markers of *K. blossfeldiana* genotypes have been recently assessed [139], allowing the selection of varieties to be used in breeding strategies. Moreover, the complete plastomes (chloroplast genomes) of five horticultural *Kalanchoe* species, *K. daigremontiana*, *K. delagoensis K. fedtschenkoi*, *K. longiflora*, and *K. pinnata*, have also been recently assembled [140]. In addition, the genome of *K. fedtschenkoi*, a species intensively used as ornamental around the world and known as a CAM model, can be currently found in the NCBI database (https://www.ncbi.nlm.nih.gov/assembly/GCA_002312845.1/ Last accessed on 25 February 2023). All these aspects allow biotechnological approaches with plants of this genus. Additionally, regarding breeding programs, new cultivars with different characteristics of flower aspects (such as color, number and shape) are being created not only by means of conventional breeding, but also by using biotechnological methods. For example, gene transformation methods have been used in *Kalanchoe*, producing plants with shorter internodes, longer-lasting flowers, and late blooming (for a deeper reading, see [141]).

Nevertheless, the knowledge and interest in the genome of *Kalanchoe* plants can also be attributed to the medicinal and commercial interest in them. In fact, understanding the enzymes involved in the biosynthesis pathway of bioactive compounds as well as the genes implicated in their biosynthesis opens new opportunities to obtain bioactive molecules at a large scale. Several recent studies have described how biotechnological approaches can be applied to increase the content of secondary metabolites in plants (in situ) or how plant cells, tissues, and organelles, as well as microorganisms (e.g., *Escherichia coli*), can be used as platforms to produce these compounds in vitro [142]. Indeed, in vitro cultures of *Kalanchoe* plants are well established. Nutrient requirements, disinfection and micropropagation protocols, as well as the establishment of suspension-cultured cells, are well known for several *Kalanchoe* species, being recently revised by García-Pérez et al. [143].

The progressive development of biotechnology led to the expansion of new strategies to produce phytopharmaceuticals and herbal medicines, creating alternatives for obtaining and producing bioactive substances [144]. In this context, plant biotechnology offers possibilities to change, in an innovative way, the metabolism of plants, increasing the production of prominent chemicals, and producing them in cells and tissues of other species [144]. Some of the main techniques used to obtain higher contents of bioactive molecules are DNA manipulation and metabolic engineering. For *Kalanchoe* plants, Fujimoto and co-workers produced novel colored-leaf plants of *K. blossfeldiana* by the ectopic expression of R2R3 MYB genes from *Tricyrtis* sp. and *Arabidopsis thaliana*, which activated the flavonoid biosynthetic pathway and led to anthocyanin accumulation [137].

Considering the most prominent bioactive molecules in *Kalanchoe*, flavonoids have more studies aiming at increasing yield or at reconstructing their biosynthetic pathways, if compared with bufadienolides. These works are interested in scaling up the obtaining of flavonoids under controlled conditions, by means of cell suspension cultures, hairy root cultures, transgenics, and microbial hosts utilization [145,146,147]. The increasing understanding of biosynthetic pathways and molecular genetics of different plants led to the higher application of metabolic engineering to enhance the production of compounds in plants and microorganisms. These achievements, however, need complete information about the biosynthetic pathway of the desired compound. The absence of such data is a barrier for several plant species [148], including *Kalanchoe*. Therefore, genomics, proteomics, and metabolomics studies in *Kalanchoe* plants are still needed and must be stimulated.

If on one hand the genetic engineering is still little applied for *Kalanchoe* plants, on the other hand, the elicitation process of the bioactive compounds in plants of the genus has been more employed and is a topic of interest in recent research. Indeed, different environmental factors can trigger the production of bioactive compounds [149], being biotechnologically applied in the development of products, including herbal and phytopharmaceuticals. For *Kalanchoe*, several elicitors have been studied and used for the enhancement of flavonoid production. In this sense, our group was the pioneer in the application of different light qualities on *Kalanchoe*. In 2010, Leal-Costa and col. demonstrated that the supplementation of blue light in the in vitro cultivation of *K. pinnata* increased the phenolic idioblasts in leaf tissues, suggesting that this light quality might stimulate the accumulation of these compounds [150]. In fact, few years later, Nascimento et al. [19] showed the effects not only of blue light but also of UV-A radiation in the enhancement of phenolics and flavonoids content as well as the antioxidant activity of *K. pinnata* extracts. The same authors also showed the elicitation of quercitrin and other flavonoids production by UV-B radiation in this same species [20]. Later, UV-B radiation was also used as elicitation strategy for the enhancement of bioactive compounds in *K. pinnata* leaves by another group, together with the biotic elicitation through natural transformation of the plants by *Agrobacterium rhizogenes* [151]. The authors observed that the combination of both factors resulted in an increase of more than 100% of the total flavonoid content [151]. Lastly, nutrient stress, salicylic acid, and cyclodextrin have also been applied as elicitors in *Kalanchoe* plants and cell suspension cultures, with the prediction of the best conditions by using new tools of machine learning [152,153,154].

## 4. Gaps of Knowledge and Challenges for Drug Development with *Kalanchoe* Compounds

Despite presenting all the potential characteristics that make *Kalanchoe* plants (and especially *K. pinnata*) important sources of bioactive molecules, some gaps of knowledge, challenges, and limitations must be presented. These are specially based on the few in vivo studies conducted with the bioactive compounds, as well as the lack of evaluation of their action mechanisms and possible toxicity. In addition, concerns about the bioavailability of these compounds and the available technological solutions ought to be mentioned. In this section, a brief critical analysis of these aspects is presented in order to contribute to future research in the field.

Generally, studies investigating bioactive substances in medicinal plants begin by demonstrating the pharmacological effects of the extracts, using in vitro and in vivo assays. While demonstrating the pharmacological activity of a plant extract is relatively simple, identifying the key constituents for this observed pharmacological effect is a challenge for researchers. The bioassay-guided fractionation of extracts focusing on a given pharmacological activity has been used as a strategy to recognize the bioactive substances, among other possibilities. Once this stage has been reached, it is essential to understand how these substances act on a given pharmacological target.

Even though the pharmacological potential of *Kalanchoe* flavonoids and bufadienolides has been evidenced by multiple studies, there is still a long way to go. For instance, only a limited number of these substances have been the object of further investigation to determine their modes of action. This is the case of the bufadienolide bryophyllin A and kalantubosides A and B, that have had their cytotoxic mechanisms assessed (Section 2.1). Regarding flavonoids, quercetin and QAR stand out due to their more in-depth mode of action studies (Section 2.2). A thorough understanding of the targets and mechanisms of action of other flavonoids and bufadienolides from *Kalanchoe* would be essential for exploring their pharmacological potential.

Regarding the type of studies conducted with *Kalanchoe* species, we can observe that most of them are in vitro assays, while in vivo studies are scarce (Table 1 and Table 2). Even though various studies have been carried out to evaluate the antitumor potential of *Kalanchoe* bufadienolides in multiple cell lineages, only one in vivo study was retrieved in the literature [29]. The same is true for the other pharmacological activities reported for these bufadienolides. There are comparatively more in vivo studies for flavonoids, but they represent only a small fraction of the research works.

Considering the use of *K. pinnata* in the treatment of wounds and inflammatory processes, there has been an important advance in the knowledge of the pharmacological role of QAR proven by in vivo assays. The antileishmanial, anti-inflammatory, antinociceptive, antiedematogenic, wound healing, and gastroprotective effects of this flavonoid were demonstrated in mice and rats [15,59,60,68,69], although there is still much to be done to clarify the intricacies of its in vivo behavior. For example, the immunomodulatory profile of QAR deserves to be further investigated considering other therapeutic targets in which the inflammatory process plays an important role. Moreover, for many other *Kalanchoe* bioactive flavonoids, there are no in vivo studies. The in vivo studies are, undoubtedly, a very important step to assess the potential of drug candidates. Promising in vitro results do not always translate into the desired in vivo effects, since the metabolism of bioactive substances is not taken into account in in vitro assays [155,156,157].

Therefore, despite the greater complexity and cost of in vivo assays, as well as the necessity to abide to stringent rules imposed by ethical considerations and regulations, animal models are still essential for the evaluation and validation of drugs [156]. In fact, assays with different animal species are required by regulatory agencies, such as the Food and Drug Administration (FDA, USA), the European Medicine Agency (EMA), and the Brazilian Health Regulatory Agency (ANVISA), before human trials are considered [156,158]. It is also important to mention that animal models frequently do not adequately predict the effects of a drug in humans. Technologies such as organ-on chip and microfluidic integrated systems, based on human cell cultures, are promising alternatives to assess the effects of bioactive compounds in complex systems [159] and could also be applied in the future for the study of flavonoids and bufadienolides from *Kalanchoe*.

Another important and often overlooked aspect regarding *Kalanchoe* bioactive compounds is toxicity. This is a concern especially for bufadienolides, which have been associated with cases of livestock intoxication after the ingestion of *Kalanchoe* plants [1,160,161]. The majority of studies with bufadienolides from *Kalanchoe* species are about their cytotoxicity against cancer cell lines; however, only in a few of them were assays also carried out with normal cell lines to compare the selectivity of the effects [25,29]. Thus, the potential toxicity risks of these bufadienolides are poorly understood.

Concerning flavonoids, even though this class of compounds has been extensively studied, toxicological assessments seem to represent just a small portion of this research. Most of the available data concerns flavonoids with a wide distribution among plants. This is the case of quercetin and kaempferol, both found in *Kalanchoe*. These flavonol aglycones have had their toxic effects assessed in multiple in vitro and in vivo studies. Although some in vitro assays point out the possible toxicity mainly due to prooxidant activity, these effects have not been confirmed in vivo. In most of these in vivo studies, quercetin and kaempferol have shown low toxicity [162,163]. On the other hand, data on the toxicity of flavonol glycosides are scarce. Many of these compounds are present in food (beverages, fruits, and vegetables) and nutritional studies have evidenced that their consumption is safe [164,165]. In fact, in vitro studies carried out by our group have shown that flavonoids from *K. pinnata* have low or even no cytotoxicity in normal cell lines [16,66]. However, studies to specifically assess the toxicological profile of *Kalanchoe* flavonol glycosides are important if these compounds are to be considered candidates for drug development.

We must also consider that bioactive substances belonging to the class of secondary metabolites are not synthesized on a large scale by plants. Their low production in plants is a challenge for the processes of extracting and purifying these compounds in order to obtain sufficient amounts to carry out pharmacological tests, especially in vivo, whether for assessing toxicity or for studying mechanisms of action in preclinical research [156]. This partially justifies the lack of studies focusing on the toxicity of the most bioactive substances already described for the *Kalanchoe* genus.

Another aspect that deserves attention is the bioavailability of the bioactive compounds. This is one of the main reasons for the failure of bioactive molecules evaluated in complex organisms, such as animal models and human individuals. In particular, bioactive substances in their original structures, although effective in the studied animal models, need to present good properties of absorption, distribution, metabolism, excretion, and low toxicity. If they do not meet these requirements, it will certainly be necessary to invest in technological formulations and/or chemical modifications, which will increase costs and time for the development of a new drug.

Bufadienolides, especially in the form of aglycones, are poorly soluble in water and have a low bioavailability [166]. Recently, Shao et al. [166] reviewed strategies to enhance these parameters, such as structural modifications, cyclodextrin inclusion, and nanodrug delivery systems. The latter were considered the most promising. Studies to better understand the bioavailability of *Kalanchoe* bufadienolides and possible strategies to improve their bioavailability are essential for their future development as drugs. Oral bioavailability is also a concern for flavonoids. It is known that flavonoid *O*-glycosides, which are major in *Kalanchoe* species, are hydrolyzed by enzymes from the intestinal tract and from the colonic microbiota, generating their aglycone counterparts, which diffuse through the intestinal epithelium [167,168]. This can represent a major drawback for glycosylated flavonoids that have shown in vitro effects. It is therefore important to evaluate the effect of their aglycone forms and/or their in vivo effects. Flavonoids also undergo hepatic metabolism, creating conjugated compounds, mostly glucuronides and sulfates. Muzitano et al. [44] evidenced that QAR and quercitrin from *K. pinnata*, after oral intake by mice, have quercetin and quercetin glucuronide as the main plasmatic metabolites. The gut microbiota can also extensively transform flavonoids, creating phenolic acid derivatives [167,168,169]. The bioavailability of *Kalanchoe* flavonoids as well as the possible effects of their metabolites are important objects for future studies. Bioavailability issues can be mitigated using multiple strategies. Examples include permeation enhancers, development of prodrugs, and nanotechnology-based formulations [167]. This topic has been extensively reviewed elsewhere [165,167,168,170,171].

Notwithstanding, the process of drug development based on bioactive substances of natural origin also requires the collaboration between the academic research sector and pharmaceutical industries, which constitutes an enormous challenge. An additional challenge is the transfer of knowledge generated in academia to the industrial sector, including the registration of intellectual property rights.

## 5. Final Considerations and Perspectives

We presented here an up to date and critical review of the most prominent bioactive compounds found in *Kalanchoe* species. Some bioactive substances present in this genus, especially in *K. pinnata*, the most studied species, are interesting from a pharmacological point of view. These compounds could be applied isolated as drugs, inspire new drugs, or even constitute part of their original plant extracts, composing a phytomedicine. In this case, knowledge of the bioactive compounds could guide and ensure the quality and efficacy of these medicines, an aspect required by some regulatory agencies worldwide.

*Kalanchoe* species are mainly a source of bufadienolides, with antitumor and muscle relaxing activities, and flavonoids, especially recognized as anti-inflammatory and wound healing agents. Among the bioactive molecules of *Kalanchoe*, the flavonoid quercetin 3-*O*-α-L-arabinopyranosyl (1→2) α-L-rhamnopyranoside (QAR), a major compound from *K. pinnata*, stands out compared to all other molecules. This flavonoid has a wide range of pharmacological evidence, including the reduction of inflammatory processes, wound healing action, and protection against gastric ulcers, all of these already proven in animal models. QAR gathers promising evidence that makes this flavonoid an excellent drug candidate for the therapy of these conditions. Moreover, being an unusual molecule present in a high quantity, QAR can be considered as the chemical and the bioactive marker of extracts and products prepared with *K. pinnata.* A significant part of the preclinical research with this flavonoid has already been carried out, but there are still important gaps to be overcome. Furthermore, taking into account the large content of QAR in the plant and the knowledge and the biotechnological tools available for the production and extraction of this molecule [20,21], the sustainable access to QAR in a sufficient quantity to develop clinical studies does not seem to be difficult to achieve.

Except for QAR, most of the other bioactive molecules described in *Kalanchoe* were only tested by in vitro assays. The few studies focusing on their in vivo effect, mode of action, and toxicity are gaps of knowledge in this field. Thus, these aspects should be considered in further studies with plants of this genus. In summary, considering the richness in bioactive compounds (some of them uncommon in nature), the easiness of propagation and cultivation of *Kalanchoe*, and the new biotechnological aspects concerning these plants, the development of new drugs with *Kalanchoe* species can be envisaged in the near future.

## Figures and Tables

**Table 1:** Antitumor activity of bufadienolides from *Kalanchoe* species.

Species	Substance	Type of Study	Cell Lines	Main Outcomes	Ref.
*Kalanchoe ceratophylla* Haw. ^1^	Kalanchoside A (**1**)	In vitro	KB and KB-VIN (nasopharyngeal), A549 (lung), 1A9 (ovarian), PC-3 (prostate), HCT-8 (colon), and A431 (epidermoid)	Cytotoxicity against all the tested cell lines with lower IC_50_ for A549 and IA9 (IC_50:_ 0.0005 and 0.0008 µg/mL)	[26]
	Kalanchoside B (**2**)	In vitro	KB and KB-VIN (nasopharyngeal), A549 (lung), 1A9 (ovarian), PC-3 (prostate), HCT-8 (colon), and A431 (epidermoid)	Cytotoxicity against all the tested cell lines with lower IC_50_ for A549 and KB (IC_50:_ 0.001 and 0.005 µg/mL)	[26]
	Kalanchoside C (**3**)	In vitro	KB and KB-VIN (nasopharyngeal), A549 (lung), 1A9 (ovarian), PC-3 (prostate), HCT-8 (colon), and A431 (epidermoid)	Cytotoxicity against all the tested cell lines with lower IC_50_ for A549 and IA9 (IC_50:_ 0.006 and 0.012 µg/mL)	[26]
	Thesiuside (**4**)	In vitro	KB and KB-VIN (nasopharyngeal), A549 (lung), 1A9 (ovarian), PC-3 (prostate), HCT-8 (colon), and A431 (epidermoid)	Cytotoxicity against all the tested cell lines with lower IC_50_ for A549 and IA9 (IC_50:_ 0.0005 and 0.002 µg/mL)	[26]
	Hellebrigenin (**5**)	In vitro	KB and KB-VIN (nasopharyngeal), A549 (lung), 1A9 (ovarian), PC-3 (prostate), HCT-8 (colon), and A431 (epidermoid)	Cytotoxicity against all the tested cell lines with lower IC_50_ for A549 and IA9 (IC_50:_ 0.001 and 0.003 µg/mL)	[26]
	Hellebrigenin-3- acetate (**6**)	In vitro	KB and KB-VIN (nasopharyngeal), A549 (lung), 1A9 (ovarian), PC-3 (prostate), HCT-8 (colon), and A431 (epidermoid)	Cytotoxicity against all the tested cell lines with lower IC_50_ for A549, KB and KB-VIN (IC_50_: 0.003; 0.008, and 0.008µg/mL)	[26]
	Bryophyllin A ^3^ (**7**)	In vitro	KB and KB-VIN (nasopharyngeal), A549 (lung), 1A9 (ovarian), PC-3 (prostate), HCT-8 (colon), and A431 (epidermoid)	Cytotoxicity against all the tested cell lines with lower IC_50_ for A549 and IA9 (IC_50_: 0.013 and 0.014 µg/mL)	[26]
	Bryophyllin B (**8**)	In vitro	KB and KB-VIN (nasopharyngeal), A549 (lung), 1A9 (ovarian), PC-3 (prostate), HCT-8 (colon), and A431 (epidermoid)	Cytotoxicity against all the tested cell lines with lower IC_50_ for A549 and IA9 (IC_50_: 0.07 and 0.20 µg/mL)	[26]
*Kalanchoe daigremontiana ×* *delagoensis*	Bersaldegenin-1,3,5- orthoacetate (**9**)	In vitro	Raji cells (lymphoblastoid cells)	100% inhibition of tumor-promoting activity by Epstein–Barr virus at 0.8 µM	[30]
	Daigremontianin (**10**)	In vitro	Raji cells (lymphoblastoid cells)	46% inhibition of tumor-promoting activity by Epstein–Barr virus at 0.8 µM	[30]
*Kalanchoe daigremontiana* Raym.-Hamet and H. Perrier	Bersaldegenin-1,3,5- orthoacetate (**9**)	In vitro	HeLa (cervical); SKOV-3 (ovarian); MCF-7 (breast) and A375 (melanoma)	Cytotoxicity against all the tested cell lines IC_50:_ 43–48 µg/mL	[25]
		In vitro	HeLa (cervical cancer)	Activity against HeLa cells through DNA-damage and cell cycle arrest	[27]
*Kalanchoe delagoensis* Eckl. and Zeyh. ^2^	Kalantuboside A (**11**)	In vitro	A549 (lung), Cal-27 (oral carcinoma), A2058 (melanoma), and HL-60 (leukemia)	Cytotoxicity against all the tested cell lines with lower IC_50_ for A2058 and HL-60 (IC_50_: 0.02 and 0.13 µM)	[31]
		In vitro	CL1-5 (lung)	IC_50_ of 70.56 ng/mL; cell death by induction of autophagy	[28]
	Kalantuboside B (**12**)	In vitro	A549 (lung), Cal-27 (oral carcinoma), A2058 (melanoma), and HL-60 (leukemia)	Cytotoxicity against all the tested cell lines with lower IC_50_ for A2058 and HL-60 (IC_50_: 0.01 and 0.04 µM)	[31]
		In vitro	CL1-5 (lung)	IC_50_ of 52.30 ng/mL; cell death by induction of autophagy	[28]
		In vitro and in vivo	A2058 (melanoma)	IC_50_: 13.00 ng/mL. Cell death by induction of apoptosis; antitumor activity in A2058-xenographted mice	[29]
	Bryophyllin A ^3^ (**7**)	In vitro	A549 (lung), Cal-27 (oral carcinoma), A2058 (melanoma), and HL-60 (leukemia)	Cytotoxicity against all the tested cell lines with lower IC_50_ for A2058 and HL-60 (IC_50_: 0.21 and 0.14 µM)	[31]
		In vitro	CL1-5 (lung)	IC_50_ of 76.70 ng/mL; cell death by induction of autophagy	[28]
	Bersaldegenin-1,3,5- orthoacetate (**9**)	In vitro	A549 (lung), Cal-27 (oral carcinoma), A2058 (melanoma), and HL-60 (leukemia)	Cytotoxicity against all the tested cell lines with lower IC_50_ for A2058 and HL-60 (IC_50_: 0.70 and 0.01 µM)	[31]
	Bersaldegenin-1- acetate (**13**)	In vitro	A549 (lung), Cal-27 (oral carcinoma), A2058 (melanoma), and HL-60 (leukemia)	Cytotoxicity against all the tested cell lines with lower IC_50_ for A2058 and HL-60 (IC_50_: 0.68 and 0.02 µM)	[31]
*Kalanchoe hybrida* Desf. ex Steud.	Bersaldegenin-3- acetate (**14**)	In vitro	MCF-7 (breast cancer), NCI-H460 (lung cancer), and SF-268 (glioblastoma)	93–100% cell growth inhibition at 4 and 20 µg/mL	[24]
	Bersaldegenin-1- acetate (**13**)	In vitro	MCF-7 (breast cancer), NCI-H460 (lung cancer), and SF-268 (glioblastoma)	69–100% cell growth inhibition at 4 and 20 µg/mL	[24]
	Daigredorigenin-3- acetate (**15**)	In vitro	MCF-7 (breast cancer), NCI-H460 (lung cancer), and SF-268 (glioblastoma)	85–100% cell growth inhibition at 4 and 20 µg/mL	[24]
*Kalanchoe pinnata* (Lam.) Person	Bryophyllin A ^3^ (**7**)	In vitro	A-549 (lung), HTC-8 (colon), and KB (nasopharyngeal)	IC_50_ of 10 to 30 ng/mL	[32]
		In vitro	Raji cells (lymphoblastoid cells)	92% inhibition of tumor-promoting activity by Epstein–Barr virus at 0.8 µM	[30]
	Bryophyllin B (**8**)	In vitro	KB (nasopharyngeal)	IC_50_ < 80 ng/mL	[23]
	Bryophyllin C (**16**)	In vitro	Raji cells (lymphoblastoid cells)	92% inhibition of tumor-promoting activity by Epstein–Barr virus at 4 µM	[30]
	Bersaldegenin-3- acetate (**14**)	In vitro	KB (nasopharyngeal), A-549 (lung), and HCT-8 (colon)	IC_50_ from 10 to 40 ng/mL	[23]
		In vitro	A549 (lung), Cal-27 (oral carcinoma), A2058 (melanoma), and HL-60 (leukemia)	Cytotoxicity against all the tested cell lines with lower IC_50_ for A2058 and HL-60 (IC_50_: 0.68 and 0.02 µM)	[31]
		In vitro	Raji cells (lymphoblastoid cells)	85% inhibition of tumor-promoting activity by Epstein–Barr virus at 20 µM	[30]

^1^ syn. *Kalanchoe gracilis* Hance; ^2^ syn: *Kalanchoe tubiflora* Raym.-Hamet; ^3^ Also known as bryotoxin C.

**Table 2:** Flavonoids from *Kalanchoe* species and their biological activities.

Species	Substance	Type of Study	Activity	Main Outcomes	Ref.
*Kalanchoe brasiliensis* Cambess. ^1^	Kalambroside A ^2^ (**19**)	In vitro	Immunosuppressive	Inhibition of human lymphocyte proliferation (IC_50_ = 0.5 µg/L)	[18]
	Kalambroside B ^3^ (**20**)	In vitro	Immunosuppressive	Inhibition of human lymphocyte proliferation (IC_50_ = 1 µg/L)	[18]
	Patuletin 3-*O*-(4″-*O*-acetyl-α-L-rhamnopyranosyl)-7-*O*-(3‴-*O*-acetyl-α-L-rhamnopyranoside) (**21**)	In vitro	Immunosuppressive	Inhibition of human lymphocyte proliferation (IC_50_ = 0.25 µg/L)	[18]
*Kalanchoe daigremontiana* Raym.-Hamet and H. Perrier	Kaempferol-3-*O*-β-D-xylopyranosyl (1→2) α-L-rhamnopyranoside (**22**)	In vitro	Antiviral	Anti-herpesvirus activity (HSV-1 and HSV-2). IC_50_ of 7.4 and 9.0 µg/mL, respectively, with high selectivity index (>20)	[11]
	Quercetin 3-*O*-β-D-xylopyranosyl (1→2) α-L-rhamnopyranoside (**23**)	In vitro	Antiviral	Anti-herpesvirus activity (HSV-1 and HSV-2). IC_50_ of 5.8 and 36.2 µg/mL, respectively, with selectivity index >5	[11]
*Kalanchoe pinnata* (Lam.) Person	Quercetin (**24**)	In vitro	Antiviral	Activity against hepatitis C virus (HCV), IC_50 =_ 6.1 μg/mL, without cytotoxicity	[63]
		In vitro/ In vivo (intragastric gavage)	Immunomodulatory	Inhibition of degranulation and cytokine production of bone marrow-derived mast cells; decrease in airway hyperresponsiveness and airway inflammation	[61]
		In vivo (oral administration)	Antileishmanial	Potent efficacy against cutaneous leishmaniasis	[60]
		In vitro	Immunosuppressive	Inhibition of lymphocyte proliferation (IC_50_ = 2.5 μg/mL)	[16]
	Quercitrin (**25**)	In vivo (oral administration)	Antianaphylactic	It prevented fatal anaphylaxis in 75% of the treated animals	[62]
		In vitro	Immunosuppressive	Moderate inhibition of lymphocyte proliferation	[16]
		In vitro	Xanthine oxidase inhibition	Moderate inhibition (124 μM) of the enzyme	[64]
		In vitro	Antioxidant	Free radical scavenging activity	[64]
		In vitro	α-glucosidase inhibition	Activity IC_50_ = 83.83 μg/mL	[65]
		In vitro/In vivo (oral administration)	Antileishmanial	Activity against *Leishmnia amazonenis* amastigotes (IC_50_~8 μg/mL); potent oral efficacy against cutaneous leishmaniasis	[60,66]
	Quercetin 3-*O*-α-L-arabinopyranosyl (1→2) α-L-rhamnopyranoside (**26**)	In vivo (subcutaneous administration)	Anti-inflammatory	Inhibition of COX-1 (IC_50_ = 22.1 μg/mL) and COX-2 (IC_50_ > 50 μg/mL).	[15]
		In silico/In vitro	Anti-inflammatory	PDE4 (phosphodiesterase 4) inhibition by acting as blocker, with high selectiveness to PDE4B	[67]
		In vitro/In vivo (oral administration)	Antileishmanial	High antileishmanial activity against *Leishmania amazonenis* amastigotes (IC_50_~45 μg/mL); potent oral efficacy against cutaneous leishmaniasis	[59,60]
		In vivo (subcutaneous administration)	Antinociceptive	Inhibition of the acetic acid-induced writhing (ID_50_ = 9.4mg/kg)	[15]
		In vitro	Immunosuppressive	Lower inhibition of lymphocyte proliferation (IC_50_ = 38.8 μg/mL)	[16]
		In vivo (topical administration)	Wound healing	Animals treated with cream containing this flavonoid exhibited 97.5 ± 0.8% of healing on the 12^th^ day of treatment	[68]
		In vitro	Antioxidant	Free radical scavenging activity	[19,64]
		In vivo (oral administration)	Gastroprotective	The pretreatment with the flavonoid (5 mg/kg) reduced gastric lesions, increased GSH, and decreased MDA levels	[69]
		In vitro	α-glucosidase inhibition	Activity IC_50_ = 110.52 μg/mL	[65]
		In vivo (subcutaneous administration)	Antiedematogenic	Reduction in the croton oil-induced ear edema (ID_50_ = 0.76 mg/kg)	[15]
	Miquelianin (**27**)	In vitro	Immunosuppressive	First time reported for *K. pinnata*, immunosuppression of murine lymphocytes	[16]
	Isoquercitrin (**28**)	In vitro	Immunosuppressive	First time reported for *K. pinnata*, immunosuppression of murine lymphocytes	[16]
	4′,5-dihydroxy-3′,8-dimethoxyflavone-7-*O*-D-glucopyranoside (**29**)	In vitro	Antileishmanial	Less active compound in comparison to the other flavonoids tested in the study IC_50_ > 100 μg/mL (>203 μM)	[59]
	Afzelin (**30**)	In vitro	Antileishmanial	First report for the antileishmanial activity of afzelin	[59]
	Kapinnatoside ^4^ (**31**)	In vitro	Antileishmanial	Less active than the quercetin glycosides	[59]
		In vitro	Xanthine oxidase inhibition	Moderate inhibition (168 μM) of the enzyme	[64]
	α-rhamnoisorobin (**32**)	In vitro	Antimicrobial	Antimicrobial activity equal to those of the positive controls; antibacterial activity (MIC = 1 μg/mL) higher than that of the ciprofloxacin against *Pseudomonas aeruginosa*	[70]
		In vitro	Antioxidant	Antioxidant activity IC_50_ = 0.71 μg/mL higher than that of the reference drug	[70]
	3′,4′-dimethoxy quercetin (**33**)	In vitro	α-glucosidase inhibition	Activity IC_50_ = 103.20 μg/mL	[65]
*Kalanchoe prolifera* Raym.-Hamet	Astragalin (**34**)	In vitro	Antitumor	Cytotoxicity against P-388 murine leukemia cells (IC_50_ = 35.9 µg/mL)	[71]
	Afzelin (**30**)	In vitro	Antitumor	Cytotoxicity against P-388 murine leukemia cells (IC_50_ = 32.1 µg/mL)	[71]
	Kaempferol (**35**)	In vitro	Antitumor	Cytotoxicity against P-388 murine leukemia cells (IC_50_ = 4.5 µg/mL)	[71]
	Quercetin (**24**)	In vitro	Antitumor	Cytotoxicity against P-388 murine leukemia cells (IC_50_ = 6.3 µg/mL)	[71]
	Isoquercitrin (**28**)	In vitro	Antitumor	Cytotoxicity against P-388 murine leukemia cells (IC_50_ = 29.1 µg/mL)	[71]
	Quercetin-3-*O*- sophoroside (**36**)	In vitro	Antitumor	Cytotoxicity against P-388 murine leukemia cells (IC_50_ = 50.4 µg/mL)	[71]
	Rutin (**37**)	In vitro	Antitumor	Cytotoxicity against P-388 murine leukemia cells (IC_50_ = 58.5 µg/mL)	[71]
*Kalanchoe tomentosa* Baker	Astragalin (**34**)	In vitro	Antitumor	Cytotoxicity against P-388 murine leukemia cells (weak activity: IC_50_ > 100 µg/mL)	[72]
	Afzelin (**30**)	In vitro	Antitumor	Cytotoxicity against P-388 murine leukemia cells (IC_50_ = 3.3 µg/mL)	[72]
		In vitro	α-amylase inhibition	Moderate inhibitory activity (IC_50_ = 379.8 μg/mL)	[73]

^1^ syn: *Kalanchoe crenata* (Andrews) Haw.; ^2^ patuletin-3-*O*-(4″-*O*-acetyl-α-L-rhamnopyranosyl)-7-*O*-(2‴-*O*-acetyl-α-L-rhamnopyranoside); ^3^ patuletin 3-rhamnoside-7-(2‴-acetylrhamnoside); ^4^ kaempferol-3-*O*-α-L-arabinopyranosyl (1→2) α-L-rhamnopyranoside.

## Data Availability

This review generated no new data. All consulted data are available on cited literature.
